# Genome sequences of three *Bartonella schoenbuchensis* strains from Czechia

**DOI:** 10.1128/mra.00397-24

**Published:** 2024-07-02

**Authors:** Luis Solis Cayo, Iva Hammerbauerová, Julian Sommer, Zahra Nemati, Wibke Ballhorn, Pablo Tsukayama, Alexander Dichter, Jan Votýpka, Volkhard A. J. Kempf

**Affiliations:** 1 Laboratorio de Microbiología Molecular y Biotecnología, Facultad de Ciencias Biológicas, Universidad Nacional Mayor de San Marcos, Lima, Peru; 2 Laboratorio de Genómica Microbiana, Facultad de Ciencias e Ingeniería, Universidad Peruana Cayetano Heredia, Lima, Peru; 3 Instituto de Medicina Tropical “Alexander von Humboldt,” Universidad Peruana Cayetano Heredia, Lima, Peru; 4 Institute for Medical Microbiology and Infection Control, University Hospital, Goethe University Frankfurt am Main, Frankfurt, Germany; 5 Department of Parasitology, Faculty of Science, Charles University, Prague, Czechia; Rochester Institute of Technology, Rochester, New York, USA

**Keywords:** *Bartonella*, *schoenbuchensis*, *Lipoptena*, genomes, Czech Republic

## Abstract

*Bartonella schoenbuchensis* causes bacteremia in ruminants and is transmitted by deer keds. Here, we report the complete genome sequences of three *B. schoenbuchensis strains* (L2, L19, and L24) recently isolated from deer keds (*Lipoptena fortisetosa*) in Czechia.

## ANNOUNCEMENT

*Bartonella schoenbuchensis* was first isolated from roe deer (1) and is transmitted by keds (*Lipoptena* spp.) (2 – 4). One case of a human infection with chronic non-specific symptoms has been reported (5). The epidemiology and infection biology of *B. schoenbuchensis* are largely unknown. Here, we report the genomic sequence of three *B. schoenbuchensis* isolates from central Europe.

*Lipoptena fortisetosa* were collected from deer shot in the Hradiště military district in Czechia in August 2023. Keds were killed by incubation at −20°C for 20 min and then washed in 70% ethanol and sterile phosphate-buffered saline. The gut was extracted and homogenized in 200 µL of *Bartonella* liquid (BaLi) medium (6) using a TissueLyser LT (Qiagen, UK), and the homogenate was cultivated on chocolate blood agar at 37°C and 5% CO_2_ for 2 weeks. From the growing bacteria, one colony from each ked was restreaked and identified by citrate synthase (*gltA*)-specific PCR (7). Bacteria were frozen in Luria-Bertani medium supplemented with 20% glycerol. Stocks were rethawed and restreaked for purity. Isolates are named after the number of the analyzed keds (e.g., L2 = *Lipoptena* isolate 2). Three *B. schoenbuchensis* isolates (L2, L19, and L24) were further analyzed.

Whole-genome sequencing (WGS) was pursued in a hybrid approach of Illumina short reads and Oxford Nanopore long reads to improve contig assembly. *B. schoenbuchensis* strains were grown in BaLi medium for 4 days at 37°C, 5% CO_2_, and 95% relative humidity. Bacteria were centrifuged (4,991 × *g*, 15 min), resuspended in Dulbecco’s phosphate-buffered saline (pH 7.0–7.3), and pelleted (4,991 × *g*, 15 min). Genomic DNA was isolated using the Qiagen DNeasy ultraclean microbial kit, sheared, and prepared for sequencing on the Novaseq platform according to the manufacturer’s instructions and sequenced on an Illumina platform (Novaseq X Plus; PE150, 1 GB raw data per sample), resulting in paired-end sequencing yields of 5,695,118 (L2), 5,349,576 (L19), and 5,804,402 (L24) read pairs. For long-read WGS, the sequencing library was prepared using the Oxford Nanopore Sequencing LSK-109 ligation sequencing protocol in combination with the native barcoding kit EXP-NBD104. The library was sequenced on Oxford Nanopore Technologies GridION instrument using R9.4.1 flowcells. Quality control of all sequencing files was performed using FastQC version 0.11.8 (7).

The genomes were *de novo* assembled and in the case of L2 closed using Flye assembler version 2.9.3 (8), polished using polypolish version 0.6.0 (9), and checked for quality using Quast version 5.2.0 (10). Genome annotation was performed using Bakta version 1.9.2 (11). For taxonomic classification, FastANI version 1.3 (12) was used against the reference genome (GCF_000385435.1). Additionally, ABRIcate version 1.0.1 (13) using the Virulence Factor Database (14) was applied to detect virulence factor genes. To refine the results, a mapping against the reference genome was employed using minimap2 version 2.28 (15), SAMtools version 1.19 (16), and medaka 1.12.0 (17), and additionally ABRIcate to detect virulence factor genes. Finally, a manual search for other genes of interest was performed using blastX (18). None of the strains harbored a detectable plasmid in the PlasmidFinder database (19). The results of the genomic sequencing and virulence factors are given in Table 1.

**TABLE 1:** Detailed analysis of the genomic sequencing results of *B. schoenbuchensis* L2, L19, and L24

	L2		L19		L24	
Taxonomic classification						
ANI to the reference genome (%)	96.0923		96.2403		96.7583	
Genome assembly and annotation						
Total length (bp)	1,669,269		1,747,515		1,751,757	
Number of contigs	1		3		5	
*N*_50_	1,669,108		1,102,263		1,252,414	
*L*_50_	1		1		1	
%GC	37.96		38.43		38.26	
Number of coding sequences	1,469		1,517		1,559	
ABRIcate results (% identity, % coverage)						
*Bartonella* adhesin A *BadA*	34.71^*a*^ 38.59^*a*^	69.79^*a*^ 69.17^*a*^	40.24	71.95	34.71^*a*^ 37.93^*a*^	69.21^*a*^ 67.72^*a*^
Type IV secretion system *virB11*	83.94	67.76	40.24	71.95	69.65	68.02
Type IV secretion system *virB9*	72.92	68.91	72.92	68.91	72.92	68.91
Type IV secretion system *virB8*	73.69	68.52	73.24	68.82	73.24	69.02
Type IV secretion system *virB4*	95.29	71.12	94.48	70.32	94.48	70.32
Type IV coupling protein *VirB/D4*	70.57	66.64	70.57	66.64	70.57	66.64
blastX results (genomic position)						
Invasion-associated locus B *ialB*	909,663–910,235		541,625–542,197		355,940–356,512	
Autotransporter *Bartonella* angiogenic factor A *BafA*	688,235–690,895		Not found		Not found	
Flagellin A *flaA*	1,543,407–1544555		636,331–637476		1,010,711–1,011,856	

^*a*^ Two *badA* homolog genes are present in the respective genome.

The following virulence factors were found: (i) trimeric autotransporter adhesin homologous to *Bartonella* adhesin A (BadA), (ii) virB/D4 type IV secretion system, (iii) *Bartonella* angiogenic factor (all homologous to that of *Bartonella henselae*), (iv) flagellin, and (v) invasion-associated locus B (IalB, both homologous to that of *Bartonella bacilliformis*) (20 – 24). Our data provide a basis for further experimental work with this emerging pathogen, e.g., to identify immunogenic determinants targeted in animal or human infections.

## Data Availability

The genome sequences have been submitted to GenBank with the following accession numbers: CP154603.1 (L2), JBCAUK000000000.1 (L19) and JBCAUL000000000.1 (L24). The associated BioProject number is PRJNA1099291. The BioSample accession numbers for long reads are SAMN40935016 (L2), SAMN40935017 (L19) and SAMN40935018 (L24) and for short reads SAMN40949163 (L2), SAMN40949164 (L19) and SAMN40949165 (L24), respectively.
